# Pomegranate seed oil protects against tacrolimus-induced toxicity in the heart and kidney by modulation of oxidative stress in rats

**DOI:** 10.22038/AJP.2022.19703

**Published:** 2022

**Authors:** Azar Hosseini, Arezoo Rajabian, Fatemeh Forouzanfar, Mahdi Farzadnia, Mohammad Taher Boroushaki

**Affiliations:** 1 *Pharmacological Research Center of Medicinal Plants, Mashhad University of Medical Sciences, Mashhad, Iran*; 2 *Department of Pharmacology, Faculty of Medicine, Mashhad University of Medical Sciences, Mashhad, Iran *; 3 *Department of Internal Medicine, Faculty of Medicine, Mashhad University of Medical Sciences, Mashhad, Iran*; 4 *Neuroscience Research Center, Mashhad University of Medical Sciences, Mashhad, Iran*; 5 *Department of Neuroscience, Faculty of Medicine, Mashhad University of Medical Sciences, Mashhad, Iran*; 6 *Cancer Molecular Pathology Research Center, Imam Reza Hospital, Faculty of Medicine, Mashhad University of Medical Sciences, Mashhad, Iran*

**Keywords:** Pomegranate seed oil, Tacrolimus, Nephrotoxicity, Cardiotoxicity

## Abstract

**Objective:**

The clinical use of tacrolimus is limited due to its side effects. This research investigated the protective activities of pomegranate seed oil (PSO) against TAC toxicity.

**Materials and Methods:**

The groups are included normal (1 ml of corn oil), TAC (2 mg/kg), and co-treatment of PSO (0.4 and 0.8 ml/kg) and TAC. All administrations were carried out intraperitoneally for 14 days. After the last injection, blood was collected from the heart.

**Results:**

TAC increased creatinine and urea. Increased malondialdehyde, reduced thiol content and superoxide dismutase. The elevation of lactate dehydrogenase, aspartate aminotransferase (AST), alanine aminotransferase (ALT), creatinine kinase-MB and creatinine phosphokinase that confirmed cardiac toxicity. PSO reduced TAC toxicity. PSO decreased TAC-induced pathology injury.

**Conclusion:**

PSO reduced TAC toxicity in renal and heart via scavenging free radicals.

## Introduction

Tacrolimus (TAC) is a macrolide agent separated from *Streptomyces tsukubaensis* (Peterka et al., 2015 ▶). It is applied as an immunosuppressive drug for inhibition of rejection of transplanted solid organ (Scalea et al., 2016 ▶). The immunosuppressive mechanism of TAC is related to prevention of calcineurin, a calcium and calmodulin-dependent phosphatase (Thomson et al., 1995 ▶). Also, TAC causes side effects in organs such as skeletal, nervous and cardiac tissues because calcineurin is found in these tissues. The calcineurin inhibition can change sympathetic activation or affect calcium release channels, therefore, it leads to neurotoxicity, nephrotoxicity, and cardiotoxicity (Atkison et al., 1995 ▶; Bechstein, 2000 ▶; Fernandes et al., 2014 ▶). The cardiotoxicity of TAC involves arteritis of cardiac arteries and extensive calcification of cardiac tissue (Atkison et al., 1997 ▶). Also, TAC causes hypertension (Roberts et al., 2002 ▶). Also, various studies have shown that it induces renal toxicity (Butani et al., 2003 ▶; Lloberas et al., 2008 ▶). The recent studies have reported that TAC increases generation of reactive oxygen species (ROS) by stimulation of the nicotinamide adenine dinucleotide phosphate oxidase pathway. However, oxidative stress may have a role in TAC nephro and cardiotoxicity (Al-Harbi et al., 2015 ▶). Different studies have shown natural products that may reduce TAC nephro and cardiotoxicity (Butani et al., 2003 ▶; Zhong et al., 2006 ▶; Lee et al., 2018 ▶). In folk medicine, pomegranate and its derivatives as pomegranate seed oil (PSO) have several pharmacological activities including antioxidant, anti- inflammatory, and anti-carcinogenic effects (Lansky and Newman, 2007 ▶), and beneficial properties against cardiovascular problems, diabetes neurological diseases, and other disorders (Hartman et al., 2006 ▶; Mena et al., 2011 ▶). The recent studies have reported that nephron- and cardio-protective effects of PSO against toxic agents were mediated via scavenging of reactive oxygen species (ROS) and lipid peroxidation due to the presence of anti-oxidant compounds (Bouroshaki, 2010 ▶, Boroushaki, 2013 ▶, Boroushaki, 2014b ▶, Boroushaki, 2015 ▶; Ahmed et al., 2016 ▶; Mollazadeh et al., 2016 ▶; Bihamta et al., 2017 ▶; Mollazadeh et al., 2017 ▶; Alkuraishy et al., 2019 ▶; Kandeil et al., 2019 ▶). In this research, we evaluated the effect of PSO on TAC nephro and cardiotoxicity.

## Materials and Methods

**Reagents**

PSO (d= 0.81 g/ml at 25C) was bought from Sarouneh company (Uromeya, Iran). n-butanol, DTNB (2,20-dinitro-5,50-dithiodibenzoic acid), TBA (2-thiobarbituric acid), Na_2_EDTA (ethylenediaminetetraacetic acid disodium salt), KCl, Trizma base [Tris(hydroxymethyl)aminomethane] HCl, phosphoric acid ((1%), methanol, TCA (trichloroacetic acid) were prepared from Merck company (Darmstadt, Germany). TAC was bought from Zahravi Pharmaceutical Company, Tabriz, Iran**.**

**Animals**

Adult male Wistar rats (200–250 g) were prepared from Laboratory Animals Research Center, Mashhad University of Medical Sciences. The temperature was controlled in the laboratory (22±4^o^C) with 12 hr dark/light cycles. The animals had free access to normal laboratory chow and tap water *ad libitum*. All laboratory activities were done according to National Institutes of Health Guide for the Care and Use of Laboratory Animals and confirmed by the Animal Ethics Committee, Mashhad University of Medical Sciences.

**Experimental protocols**

The animals were divided into 4 groups of six each. The rats received intra-peritoneal (i.p) of TAC alone and in combination with PSO as follows: The corn oil was administered to control group for 14 days. The 2^nd^ group received TAC 2 mg/kg for 14 days. PSO was injected at doses of 0.4 and 0.8 ml/kg (Mollazadeh et al., 2017) with TAC (2 mg/kg) (Al-Harbi et al., 2015 ▶) for 14 days , respectively to the 3^rd^ and 4^th^ groups. Twenty-four hours after the last treatment, ketamine (60 mg/kg) and xylazine (10 mg/kg) were used to anesthetize the rats. Blood samples were collected by cardiac puncture to determine biochemical indexes. The serum was isolated via centrifugation at 1800 g for 10 min and kept at freezer. The kidneys and heart were isolated. The heart was halved, one kidney and a half of heart were fixed in formalin, dehydrated and finally, sectioned for histopathological evaluations. The other half of heart and the other kidney were used for measurement of malondialdehyde and thiol content. 

**Biochemical analysis**

**Determination of urea and creatinine in serum**

The level of creatinine and urea in serum shows the function of kidney. These parameters were evaluated by urea kit prepared from Man Lab Company, Tehran, Iran. The Jaffe’s protocol was used to determine the concentration of creatinine in serum (Junge et al., 2004 ▶).

**Measurement of lipid peroxidation**

The main product of lipid peroxidation is malondialdehyde (MDA) which makes a red color when reacts with TBA, producing a peak absorbance at 532 nm (Hosseinzadeh et al., 2005 ▶). In centrifuge tubes, 0.5 ml homogenate was mixed with 3 mL phosphoric acid (1%) and 1 ml TBA (0.6%). All tubes were put in a boiling water bath for 45 min. Then, the tubes were cooled and 4 ml n-butanol was added to the reaction mixture, vortexed for 1 min, and centrifuged at 20,000 rpm for 20 min. The absorbance of the organic layer was measured at 532 nm. Amount of MDA is reported as nanomoles per gram of tissue. MDA concentration in the kidney homogenates was calculated using the standard curve of MDA (concentration range 0–40 mM). 

**Measurement of sulfhydryl groups**

The content of sulfhydryl (SH) group was measured in homogenates of the kidney and heart spectrophotometrically using DTNB as a coloring reagent. When DTNB bonds with sulfhydryl leads to yellow color. Here, 50 μl of homogenate was mixed with 1 ml Tris EDTA buffer in test tube (pH 8.6) and absorbance was read at 412 nm (A1); then, 20 µl of 10 mM DTNB was added. After 15 min at room temperature, the absorption was measured again (A2). Blank (B) was the absorbance of DTNB reagent. Total SH groups level was calculated using the following equation: 

Total thiol concentration mM=(A2-A1-B) *1.07/0.05*13.6

**Determination of heart function indexes**

After blood collection, serum was separated by centrifugation at 4℃ at the speed of 3000 rpm for 10 min. The activity of AST, lactate dehydrogenase (LDH), creatinine phosphokinase (CPK) and creatinine kinase-muscle brain (CKMB) in serum samples was determined by Elisa kit according to the manufacturer's guidance.

**Superoxide dismutase (SOD) activity**

The Madesh and Balasubramanian method was applied to measure the activity of SOD. The production of superoxide was measured by pyrogallol auto-oxidation and inhibition of superoxide-dependent reduction of the tetrazolium dye to its formosan by SOD was determined at 570 nm. A unit of SOD activity is defined as the amount of enzyme that induces 50% inhibition in the MTT reduction rate (Madesh and Balasubramanian, 1998 ▶).

**Histopathological examination**

The heart and kidney of rats were fixed in 10% formalin, processed in different graded alcohol concentrations, embedded in paraffin, and sectioned for histopathological evaluations. Hematoxylin and eosin were used to stain the sections of tissues and samples were observed under a light microscope.

**Statistical analysis**

 Data is expressed as mean±SEM. Statistical analysis was performed using Prism 6 software (La Jolla, CA). Data was analyzed using one-way analysis of variance (ANOVA) followed by the Tukey-Kramer *post-hoc* test for making comparisons between groups. P-values less than 0.05 were considered statistically significant.

## Results

**Effect of PSO on serum creatinine and urea**

**Figure1:**
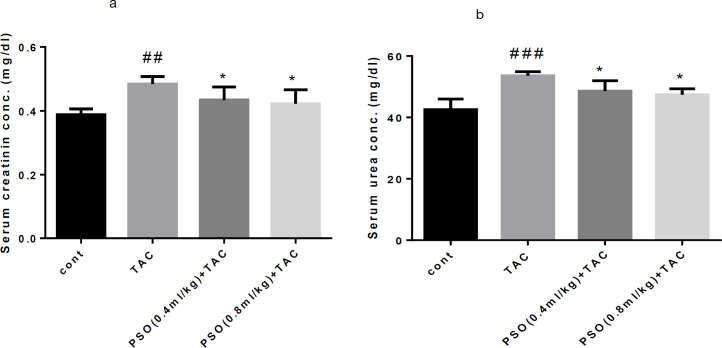
Effect of tacrolimus (TAC) and pomegranate seed oil (PSO) alone and combined on serum creatinine and urea (a, b). Data are expressed as mean±SEM; ^*^p<0.05, significant difference from the TAC; ^##^p<0.01 and ^###^p<0.01 significant difference from the control (cont) group

As shown in Figure 1a, TAC affected renal function as reflected by elevation of serum creatinine in comparison with the control group (p<0.01) (Figure 1a). The administration of PSO at both doses, decreased significantly serum creatinine in comparison with the TAC group (p<0.05) (Figure 1a). Also, serum urea increased by TAC (p<0.001) while PSO significantly attenuated it (p<0.05) in comparison with the TAC group (Figure1b). 

**Effect of PSO on TAC-induced oxidative damage in the kidney and heart**

**Figure 2:**
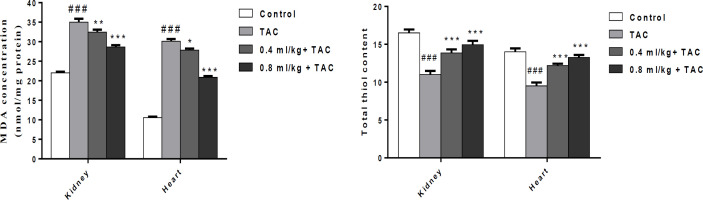
Effect of tacrolimus (TAC) and pomegranate seed oil on lipid peroxidation and thiol content in the kidney and heart. Data are expressed as mean±SEM; ^*^p<0.05, ^**^p<0.01 and ^***^p<0.001, significant difference from the TAC; and ^###^p<0.01 significant differences from the control group

The obtained results showed that TAC increased MDA as lipid peroxidation index in the heart and kidney (p<0.001) (Figure 2). As shown in Figure 1, PSO at both doses decreased the level of MDA in the kidney (0.4 ml/kg p<0.01; 0.8 ml/kg p<0.001) and heart (0.4 ml/kg p<0.05; 0.8 ml/kg p<0.001) (Figure 1a). In comparison with the control group, TAC decreased the content of thiol significantly (p<0.001) in the heart and kidney while PSO at both doses attenuated the content of thiol in both tissues (p<0.001) in comparison with the TAC group (Figure 2).

**Effect of PSO on cardiac injury markers**

**Figure 3:**
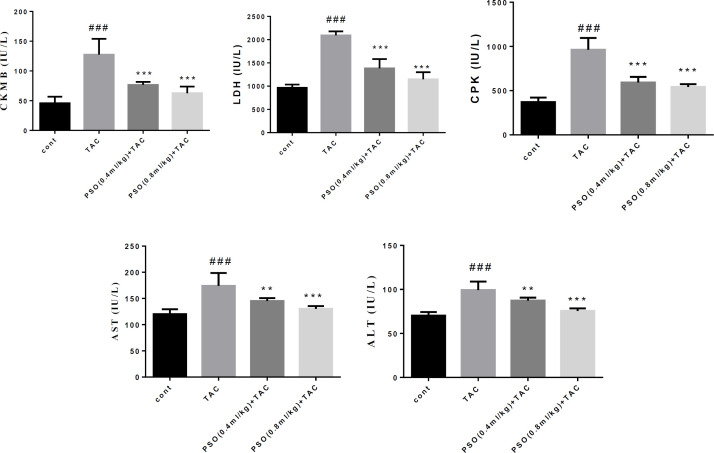
Effect of tacrolimus (TAC) and pomegranate seed oil on creatinine kinase-muscle brain (CK-MB), lactate dehydrogenase (LDH), creatinine phosphokinase (CPK), aspartate aminotransferase (AST) and alanine aminotransferase (ALT) in the heart. Data are expressed as mean±SEM; ^**^p<0.01 and ^***^p<0.001 significant difference from the TAC and ^###^p<0.01 significant difference from the control group

Our findings showed TAC increased significantly CK-MB, LDH, CPK, AST and ALT in serum (p<0.001). PSO decreased the concentration of CK-MB, LDH and CPK at both doses of PSO (p<0.001) compare to TAC group. Also, PSO attenuated significantly AST and ALT (0.4 mg/kg (p<0.01); 0.8 mg/kg (p<0.001) (Figure 3a-e).

**Effect of PSO on SOD activity**

**Figure 4:**
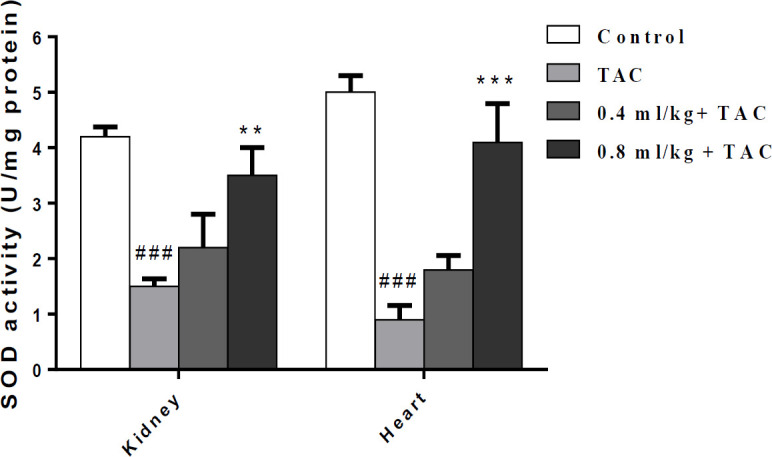
Effect of tacrolimus (TAC) and pomegranate seed oil (PSO) on SOD activity in the kidney and heart. Data are expressed as mean±SEM; ^**^p<0.01 and ^***^p<0.001 significant differences from the TAC and ^###^p<0.01 significant differences from the control group

As shown in Figure 4, TAC reduced the activity of SOD as an anti-oxidant enzyme significantly in the heart and kidney in comparison with the control group (p<0.001). Co-administration of PSO 0.8 ml/kg could reduce TAC-induced oxidative stress via increasing SOD activity in the kidney (p<0.01) and the heart (p<0.001) in comparison with the TAC group. The elevation of SOD activity by PSO 0.4 ml/kg was not significant in comparison with the TAC 

group. 

**Histopathological modifications in the kidney and heart**

**Figure 5:**
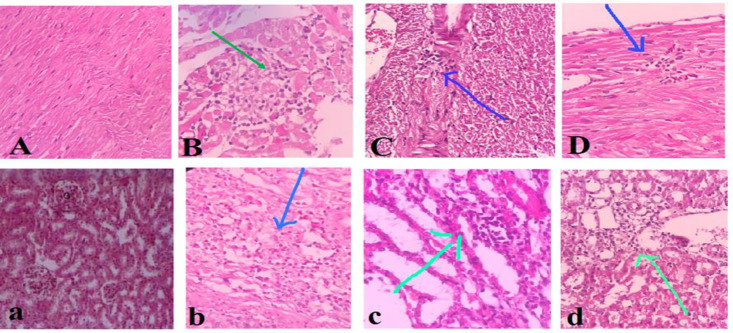
Effect of pomegranate seed oil on histopathological changes in the heart and kidney. A (×4) and a (×10): normal structure of the heart and kidney, B (×40) and b (×10): Tacrolimus group of heart and kidney, C (×40) and c (×4): TAC+PSO (0.4 ml/kg) of heart and kidney, D (×10) and d (×10): TAC+PSO (0.8 ml/kg) of heart and kidney homogenates. Arrows show damage (necrosis) to different parts of kidney and heart

The microscopic examinations showed normal structure of the kidney and heart in the control group (Figure 5). Muscle degeneration and mild chronic inflammation in the heart in the TAC group, were observed. Mild chronic inflammation of myocardium was shown in groups treated with either dose of PSO (0.4 and 0.8 ml/kg) (Figure 5A-D). Also, TAC caused acute sinus inflammation in the kidney. The PSO treated groups (0.4 and 0.8 ml/kg) revealed mild focal inflammation in the kidney. As shown in Table1, administration of PSO at both doses reduced TAC-induced toxicity in the heart and kidney.

**Table 1:** Histopathological scores in various groups

Tissue	Control group	TAC group	PSO (0.4 ml/kg)	PSO (0.8 ml/kg)
Kidney	0	+++	++	+
Heart	0	+++	++	+

TAC: Tacrolimus

PSO: Pomegranate seed oil

## Discussion

TAC as a calcineurin inhibitor is administered to recipients of solid organ transplants, but it leads to cardio and renal toxicity (McLeod et al., 2017 ▶; Park et al., 2018 ▶). The main factor in TAC toxicity is generation of ROS and inflammation which finally leads to apoptosis (Roberts et al., 2002 ▶; Kidokoro et al., 2012 ▶). The recent studies have reported that various compounds can reduce TAC-induced toxicity in the kidney (Luo et al., 2019 ▶) and in the heart (Ferjani et al., 2017 ▶) via modulation of oxidative stress. Nowadays, the researchers have high attention to medicinal plants duo to lower side effects and higher availability (Taylor et al., 2001 ▶). Also, phytochemical compounds are composed of various ingredients which have anti-oxidant and anti-inflammatory properties (Tiwari and Husain, 2017 ▶). 

Different studies have reported cardio-protective (Basu and Penugonda, 2009 ▶; Asgary et al., 2013 ▶; Al-Kuraishy and Al-Gareeb, 2016 ▶) and nephro-protective (El-Habibi, 2013 ▶; Boroushaki et al., 2016 ▶; Al-Kuraishy et al., 2019 ▶) effect of pomegranate fruit or pomegranate seed oil (PSO). The effect of PSO against TAC-induced cardio and renal had not been evaluated. Therefore, this research was designed to investigate cardio and nephro protective effect of PSO against TAC. 

Our results showed that PSO had positive effects in reduction of TAC injuries in the kidney and heart. TAC increased the concentration of creatinine, urea, AST, ALT, LDH, CKMB and CPK. The observed results were confirmed by recent studies (Al-Harbi et al., 2014 ▶; Ferjani et al., 2016 ▶). Administration of pomegranate fruit extract at 600 mg/kg/day for 90 days and PSO at dose of 50000 ppm for 30 days to rats did not show any toxicity (Patel et al., 2008 ▶; Meerts et al., 2009 ▶). 

Co-treatment with PSO and TAC attenuated creatinine, urea, ALT, AST, CPK, LDH and CKMB significantly. Nephroprotective effect and reduction of creatinine by PSO have been reported in other studies (Bouroshaki, 2010 ▶; Boroushaki, 2013 ▶; Boroushaki, 2014a ▶; Boroushaki, 2014b ▶; Boroushaki, 2015 ▶). The elevation of LDH, AST, ALT and CKMB in serum indicated cardio myocytes injury (Afsar et al., 2017 ▶). Enzyme leakage occurs due to increased levels of free radicals which caused degradation of myocyte structure (Afsar et al., 2017 ▶). Some studies have reported decreased cardiac enzymes following PSO administration (Khalil, 2004 ▶; Mollazadeh et al., 2016 ▶). We determined lipid peroxidation in in the heart and kidney as a measure of oxidative stress. Our results showed that TAC increased the level of MDA which was reversed by PSO. The elevation of MDA can be related to ROS production following TAC administration (Długosz et al., 2007 ▶). SOD acts as catalyzer for conversion of superoxide radicals to hydrogen peroxide (Jalilov et al., 2016 ▶). The excess generation of ROS may be lead to decreasing of SOD activity. In the present study, the activity of SOD was measured in these tissues. TAC decreased the activity of SOD which is accordance with recent reports (Han et al., 2006 ▶). The results showed that co-treatment with PSO and TAC increased SOD activity and thiol content. However, the anti-oxidative activity of PSO (Gil et al., 2000 ▶) has an important role in reduction of TAC -induced toxicity. Histopathological studies reveled that TAC induces toxicity in the heart as muscle degeneration and mild chronic inflammation while PSO reduced TAC cardiotoxicity and caused lower chronic inflammation of myocardium at both doses. Renal toxicity is observed as  acute sinus inflammation in the kidney in the TAC group while in treatment groups (both doses of PSO) a mild focal inflammation in the kidney was observed.

In conclusion, this research revealed that the progression of TAC-induced kidney and cardiac changes could be inhibited or attenuated by PSO. The nephro-protective and cardio-protective effect of PSO may be mediated via free radical scavenging effects. More investigations are needed to identify other mechanisms. 

## Conflicts of interest

The authors have declared that there is no conflict of interest.
